# Reviewing the review: a qualitative assessment of the peer review process in surgical journals

**DOI:** 10.1186/s41073-018-0048-0

**Published:** 2018-05-23

**Authors:** Catherine H. Davis, Barbara L. Bass, Kevin E. Behrns, Keith D. Lillemoe, O. James Garden, Mark S. Roh, Jeffrey E. Lee, Charles M. Balch, Thomas A. Aloia

**Affiliations:** 1 Department of Surgical Oncology, The University of Texas MD Anderson Cancer Center, 1400 Herman Pressler, Unit 1484, Houston, TX 77030 USA; 2 Department of Surgery, Houston Methodist Hospital, Houston, TX USA; 3 Department of Surgery, Saint Louis University School of Medicine, Saint Louis, MO USA; 4 Department of Surgery, Massachusetts General Hospital, Boston, MA USA; 5 Royal Infirmary of Edinburgh, Edinburgh, UK; 6 Orlando Health, The University of Florida Health Cancer Center, Orlando, FL USA

**Keywords:** Surgery journals, Surgical research, Surgical manuscripts, Manuscript review, Journal reviewer

## Abstract

**Background:**

Despite rapid growth of the scientific literature, no consensus guidelines have emerged to define the optimal criteria for editors to grade submitted manuscripts. The purpose of this project was to assess the peer reviewer metrics currently used in the surgical literature to evaluate original manuscript submissions.

**Methods:**

Manuscript grading forms for 14 of the highest circulation general surgery-related journals were evaluated for content, including the type and number of quantitative and qualitative questions asked of peer reviewers. Reviewer grading forms for the seven surgical journals with the higher impact factors were compared to the seven surgical journals with lower impact factors using Fisher’s exact tests.

**Results:**

Impact factors of the studied journals ranged from 1.73 to 8.57, with a median impact factor of 4.26 in the higher group and 2.81 in the lower group. The content of the grading forms was found to vary considerably. Relatively few journals asked reviewers to grade specific components of a manuscript. Higher impact factor journal manuscript grading forms more frequently addressed statistical analysis, ethical considerations, and conflict of interest. In contrast, lower impact factor journals more commonly requested reviewers to make qualitative assessments of novelty/originality, scientific validity, and scientific importance.

**Conclusion:**

Substantial variation exists in the grading criteria used to evaluate original manuscripts submitted to the surgical literature for peer review, with differential emphasis placed on certain criteria correlated to journal impact factors.

## Background

The first known incidence of peer review in the biomedical literature was performed by the Royal Society of Edinburgh in 1731, but the practice was not extensively adopted until the second half of the twentieth century [1]. Even then, the transition was far from organized and widely resisted by editors because of the low supply to demand ratio of medical articles [2]. Many medical journals first appointed small advisory boards and committees to perform “internal reviews” before transitioning to a more modern model of external peer review [2]. Over the course of the twentieth century, the number of medical articles submitted to journals exceeded demand and has continued to exponentially grow. As of 2012, there were 2 million peer reviewed articles published annually by 28,000 scholarly journals [1]. In the current era, most of the scientific, including surgical, literature is evaluated and published via a peer review process.

While an optimal peer review process aims to maintain a high level of research integrity and a literature that supports the practice of evidence-based medicine, these systems are imperfect [1, 3]. Unfortunately, not all peer review is currently performed to a high standard, and there is concern that manuscripts published after inadequate peer review will negatively affect future literature reviews, meta-analyses, and most importantly, medical practice [1, 4]. Where there are additional hurdles to collection of high quality evidence in the form of randomized studies, less rigorous research could adversely affect surgical patient care [5].

Over the past 10 years, large strides have been made in creating clear and widely accepted guidelines for authors performing studies and writing manuscripts, specifically by formatting the reporting of various study designs [4–9]. Despite supposed widespread acceptance of such guidelines, adoption has been poor in the surgical literature [10]. In contrast, no consensus guidelines have been emerged to define the optimal criteria for editors to grade the peer reviewed literature. The only standardized guidelines for performing scientific peer review were published by the Council of Biology Editors (CBE, now the Council of Science Editors) in 1983 [11]. These guidelines emphasized grading specific manuscript components, including scientific importance, clarity, study design and methods, validity of interpretation and statistical methods, appropriate literature review, written presentation, and quality. The CBE recommendations were adapted and expanded by Frank in 1996, adding that reviewers should disclose conflicts of interest and editors should provide comprehensive instructions to reviewers, with an emphasis on training first-time reviewers [12]. Likewise, the only published guidelines for peer review that are specific to surgery [13–15] focus on educating student and novice peer reviewers on the process and largely mirror the CBE recommendations with two [13, 15] including more comprehensive instruction on proper reviewer report writing.

In the absence of clear consensus guidelines, editorial boards have evolved unique and variable styles for the grading of surgical manuscripts. To determine the degree of variability and any associations between grading components and journal impact factor, this project was designed to assess the current metrics used in the surgical literature to evaluate submitted original manuscripts.

## Methods

Manuscript grading forms for 14 of the highest circulation general surgery-related journals were collected from journal editors and peer reviewers (*Annals of Surgery*, *Annals of Surgical Oncology*, *BJS* (formerly the *British Journal of Surgery*), *Digestive Surgery*, *European Journal of Surgical Oncology*, *HPB*, *JAMA Surgery*, *Journal of the American College of Surgeons*, *Journal of Gastrointestinal Surgery*, *Journal of Surgical Oncology*, *Journal of Surgical Research*, *Surgery*, *Surgical Endoscopy*, and *World Journal of Surgery*). Journals were ranked in quality as determined by journal impact factor (2015, Thomson Reuters [16]). Each grading form was evaluated for content, including the type and number of specific quantitative and qualitative questions asked of the peer reviewer. Every question contained in the grading forms was recorded and categorized. This analysis included identification of specific questions related to the following: recommendation for publication/further steps, overall manuscript rating, manuscript priority, need for statistical review, comments to author and editor, questions about specific manuscript components (abstract, background, methods, statistics, results, discussion), ethical issues, figures/tables, references, written presentation/grammar, conflict of interest, novelty/originality, clinical importance/relevance, scientific importance, and scientific validity. The inclusion of specific question types on reviewer grading forms was compared between the seven surgical journals with the higher impact factors and the seven surgical journals with lower impact factors using Fisher’s exact tests. A two-tailed *p* < 0.05 was considered statistically significant. All statistical analyses were performed using SPSS software (version 23.0, IBM Corp).

## Results

Overall, the median impact factor for the cohort of 14 journals included in the study was 3.25 (range 1.73–8.57). For the group of higher impact factor journals, the median impact factor was 4.26 (range 3.18–8.57), and for the group of lower impact factor journals, the median impact factor was 2.81 (range 1.73–3.15). Substantial variability in the distribution of the total number of questions was observed between journal groups. High impact factor group journals asked a median 11 questions (range 5–26) and lower impact factor journals asked a median of 10 questions (range 3–14). None of the 14 journals conducted blinded review (i.e., reviewers were aware of authorship at the time of their review). One of the 14 journals offered the reviewer the option of unblinding their name to the authors, but none mandated disclosure of the reviewer’s name to the authors.

The content of the grading forms was also found to vary considerably. (Table 1). The only two questions asked by all 14 journals were overall recommendation and comments to the editor. All 7 of the higher impact journals solicited comments to the authors. Relatively few journals asked reviewers to grade-specific components of a manuscript, including the abstract, introduction, methods, results, discussion, figures and tables, references, ethical issues/IRB, and written presentation and grammar (range 2–5 out of all 14 journals). Interestingly, no lower impact journals included questions about the need for further statistical review, ethical issues, or reviewer conflict of interest.

**Table 1:** Surgical manuscript review questions stratified by the journal impact factor

Question type	All journals	Higher impact journals	Lower impact journals	*P* value
Rating questions				
Recommendation	14/14	7/7	7/7	ND
Overall manuscript rating	4/14	2/7	2/7	ND
Manuscript priority	6/14	4/7	2/7	0.592
Statistical review required	5/14	5/7	0/7	0.021
Comments to author	13/14	7/7	6/7	1.000
Comments to editor	14/14	7/7	7/7	ND
Quantitative questions/specific manuscript components				
Abstract	2/14	1/7	1/7	ND
Introduction	3/14	1/7	2/7	1.000
Materials and methods	3/14	1/7	2/7	1.000
Ethical issues/IRB approval	3/14	3/7	0/7	0.192
Analysis/results	3/14	1/7	2/7	1.000
Discussion and conclusions	3/14	1/7	2/7	1.000
Figures/tables	4/14	2/7	2/7	ND
References	3/14	1/7	2/7	1.000
Written presentation/grammar	5/14	2/7	3/7	1.000
Conflict of interest	2/14	2/7	0/7	0.462
Qualitative questions				
Novelty/originality	7/14	2/7	5/7	0.286
Clinical importance/relevance	3/14	2/7	1/7	1.000
Scientific importance	7/14	3/7	4/7	1.000
Scientific validity	5/14	2/7	3/7	1.000

*ND* no difference

Higher impact factor journal manuscript grading forms more frequently addressed these issues, including statistical analysis (5/7 vs. 0/7, *p* = 0.021). Though not reaching statistical significance, possibly secondary to small group size, ethical considerations (3/7 vs. 0/7), and conflict of interest (2/7 vs. 0/7) trended to favor higher impact journals. In addition, higher impact factor journals tended to more commonly ask the review to assign a manuscript priority (4/7 vs. 2/7). In contrast, lower impact factor journals tended to more commonly request reviewers to make qualitative assessments of novelty/originality (5/7 vs. 2/7). Lower impact factor journals also asked more often about specific manuscript sections such as the abstract, introduction, methods, results, and discussion.

## Discussion

Although this study is limited by a small sample size, it did include widely circulated, English-language general surgery journals. The main findings of the study are that there is a significant variability in the specific criteria that surgical journals use to evaluate submitted original manuscripts and that the use of certain criteria is associated with impact factor levels. These data are congruent with previous literature on this topic, finding agreement on the wide variability in peer review structure [1, 4, 12, 17, 18].

Despite multiple critiques of the current system [3, 4, 12, 18, 19], implementation of consensus guidelines for standardization and optimization of the peer review process continues to be lacking. In this study, multiple of the specific points from the guidelines published by the CBE [11] and Frank [12] were identified in the reviewer grading forms, but there was considerable inconsistency between different journals, and almost none addressed the majority of the suggested guidelines.

Regarding previous studies aimed at assessing the peer review process, two similarly designed but non-surgical and now dated studies have been previously reported in the scientific literature. First, Frank in 1996 studied the review forms, instructions for reviewers, and cover letters of 67 out of the top 100 journals rated by the 1989 Institute for Scientific Information citation frequency index [12]. As in the current study, Frank found great variability in the instructions and questions asked of reviewers. Only 25% of journals provided reviewer instructions longer than one page in length. Few journals asked for assessments of soundness and quality (29%) and ethical issues (36%) or reminded reviewers of confidentiality (46%). Half of the journals solicited assessments of manuscript conclusions (51%) or appropriateness of the manuscript for the particular journal (51%). Many journals asked for assessment of manuscript novelty (72%) and priority/importance/significance (88%). Almost all journals asked reviewers to recommend manuscript acceptance or rejection (96%). Second, Weller and colleagues in 1990 compared the peer review process between 16 higher impact medical journals and 73 lower impact medical journals and found that lower impact journals were more likely to use peer reviewers in important decision points in the publication process than higher impact journals, which relied more on their editorial staff [17]. Lower impact journals were also more likely than higher impact journals to conduct blind review. Though this study provided an important snapshot of the peer review process, no reviewer feedback was collected as part of this study.

The issue of differences in the review process of journals with similar topic/focus areas (e.g., general surgery) has two sides. On the one hand, the data indicate that there may be room for improvement through standardization of the review process of the surgical literature. Although not providing concrete guidelines, previous authors have made suggestions for improvement of the peer review process, including formal training of reviewers by the journal [18, 19], standardization and creation of manuscript review protocols [12, 18], recruiting peer reviewers in the same niche field addressed in the manuscript [4], recognizing and treating peer review as a professional skill which is rewarded [3, 4, 18], editorial review of reviewer quality with feedback on performance to reviewers [18], and blinding of reviewers [18]. Given that the current study demonstrated none of the 14 top impact factor general surgery journals had a blinded review process, this could be an important advance in the surgical literature. There is mixed evidence in the literature supporting [20, 21] versus invalidating [22, 23] the importance of blinded peer review. However, it is possible that, as researchers in the same subspecialties have various professional relationships and in some cases may be competing for various grants or positions, non-blinded review may create significant bias [4].

On the other hand, variability in peer review grading criteria allows a journal and its editorial board to differentiate the focus and content of their publications, targeting the needs of their specific readership. Higher impact factor journals may justifiably place a greater emphasis on novelty and importance. Thus, while some basic criteria may be important for all journal review forms, there may be good reason to allow for diversity of emphasis or additional criteria. Along the same lines, rigid adherence to a relatively large number of criteria or check boxes, especially for an inexperienced reviewer, risks the reviewer missing the “forest for the trees.” Furthermore, editors may find the narrative detailed review in prose submitted by the reviewer to be more valuable than a quantitative scoring of individual manuscript components.

In order to reconcile these arguments that favor continued variability in the peer review grading process, it may be incumbent on editors to become more transparent to authors and readers about their journal’s unique areas of emphasis (Table 2). Currently, the peer review process, including the selection of reviewers, is incredibly variable between journals and is rarely transparent to the authors or the journal readership [1, 3, 12]. While different journals may necessarily emphasize different aspects in manuscript grading based on journal focus, process variability could also indicate that some journals are missing important specific elements of the review process that could improve the scientific quality of their publications [12].

**Table 2:** Suggestions for improvement of the manuscript review process in surgery

1) Blinding reviewers to authors	
2) At least voluntary unblinding of reviewers to authors	
3) Continued emphasis on quantitative scoring of key manuscript elements including statistical validity and ethical concerns	
4) Transparency in unique criteria of emphasis to reviewers and authors	
5) Guidelines for structuring narrative comments to editors and authors [13]	

The major concern regarding variability in the peer review process is the potential for this variability to introduce bias into the system. Peer review bias is an important problem in the scientific publication process. Peer review can be politically charged and bias, which can be positive or negative, can be directed for or against specific authors, fields, or institutions, resulting in publication bias and/or prolonged delays in publication time [1, 19]. Even as new markets open up within countries, fields, and the internet, as the academic credit toward promotion and tenure awarded for participation as an ad hoc reviewer to journals has declined, the available cohort of willing reviewers is shrinking [4]. This contraction of the reviewer pool is occurring even as the volume of submissions to surgical journals is increasing, particularly from international authorship groups [4]. Development of reviewer education programs and structured review forms that expedite the workload of reviews may encourage reviewers to stay engaged with the process and stave off the increasing pressure on editors to administratively reject submissions without peer review.

The data from this analysis suggest that overall variability and specific variability associated with impact factor may be limiting the overall quality in the surgical literature. While maintaining focus areas specific to a given journal, there may be an opportunity to increase uniformity and transparency of grading criteria through the acceptance of previously published guidelines. As there is no existing organization that brings together all of the editors of surgical journals, an organization such as the American College of Surgeons or the American Board of Surgery, which oversee all surgical specialties, may be well positioned to recommend standardized review practices to impact the quality of the surgical literature. Future specific areas for improvement of peer review of the surgical literature may include standardization of the grading form, blinding of reviewers to authors, exposure of reviewer identity to authors, and increased transparency of the review process to submitting authors (Table 2).

## Conclusions

Substantial variation exists in the grading criteria used to evaluate original manuscripts submitted to the surgical literature for peer review, with differential emphasis placed on certain criteria correlated to journal impact factors. By directly comparing the review processes of similar journals, it is apparent that potential gaps exist in the review process and that the surgical literature may benefit from a more uniform template for literature peer review.

## Abbreviations

CBE: Council of Biology Editors
